# Luteolin Attenuates Hypoxia-Induced Ferroptosis in Human Brain Microvascular Endothelial Cells Through Modulation of the PPARγ/FABP5/ALOX15 Signaling Pathway

**DOI:** 10.3390/biomedicines14091981

**Published:** 2026-09-02

**Authors:** Sirirak Mukem, Patcharakorn Kiatamornrak, Saowaros Suwansa-ard, Tipsuda Thongbuakaew

**Affiliations:** 1 School of Medicine, Walailak University, Nakhon Si Thammarat 80160, Thailand; 2 Excellence Center for Critical Care Nephrology, King Chulalongkorn Memorial Hospital, The Thai Red Cross Society, Bangkok 10330, Thailand; 3 Center of Excellence in Critical Care Nephrology, Chulalongkorn University, Bangkok 10330, Thailand; 4 School of Science, Technology and Engineering, University of the Sunshine Coast, Maroochydore, QLD 4558, Australia

**Keywords:** ferroptosis, luteolin, hypoxia, oxidative stress, endothelial dysfunction, lipid peroxidation, neuroprotection, blood–brain barrier

## Abstract

**Background:** Hypoxia-induced oxidative stress and ferroptosis contribute to blood–brain barrier dysfunction and neurovascular injury associated with ischemic stroke and neurodegenerative diseases. Luteolin, a naturally occurring flavonoid with potent antioxidant and anti-inflammatory activities, has emerged as a potential neuroprotective agent; however, its effects on hypoxia-induced ferroptosis in brain microvascular endothelial cells remain unclear. This study investigated the protective effects of luteolin and its association with changes in the PPARγ/FABP5/ALOX15 signaling pathway during hypoxia-induced ferroptotic injury. **Methods:** Human brain microvascular endothelial cells (HBEC-5i) were exposed to cobalt chloride (CoCl_2_) to establish an in vitro hypoxia model and subsequently treated with luteolin. Cell viability was assessed using the MTT assay. Intracellular Fe^2+^ accumulation, reactive oxygen species (ROS), malondialdehyde (MDA), and glutathione (GSH/GSSG) levels were determined using biochemical assays. The expression of ferroptosis- and hypoxia-related proteins, including GPX4, ALOX15, FABP5, PPARγ, HIF-1α, and VEGF, was evaluated by Western blotting. **Results:** CoCl_2_-induced hypoxia significantly reduced cell viability and increased intracellular Fe^2+^ accumulation, ROS generation, lipid peroxidation, and ferroptosis-associated signaling in HBEC-5i cells. Hypoxic conditions upregulated ALOX15 and FABP5 expression while suppressing GPX4 and PPARγ levels. Luteolin treatment markedly attenuated these effects by restoring GSH/GSSG homeostasis, enhancing GPX4 expression, reducing ALOX15-mediated lipid peroxidation, and suppressing HIF-1α and VEGF expression. In addition, luteolin modulated the PPARγ/FABP5 signaling pathway, suggesting its involvement in the regulation of hypoxia-induced ferroptosis. **Conclusions:** Luteolin attenuates hypoxia-induced ferroptosis-associated changes in human brain microvascular endothelial cells by restoring redox homeostasis and is associated with coordinated modulation of the PPARγ/FABP5/ALOX15 pathway. These findings extend previous reports of the anti-ferroptotic effects of luteolin by providing evidence in brain microvascular endothelial cells under hypoxia-mimetic stress.

## 1. Introduction

Ferroptosis is an iron-dependent form of regulated cell death characterized by the iron-regulated accumulation of lipid-based reactive oxygen species (ROS), inactivation of glutathione peroxidase 4 (GPX4), and subsequent oxidative degradation of membrane-bound polyunsaturated fatty acids (PUFAs) [1,2]. Ferroptosis involves genetic, metabolic, and protein-mediated regulatory, triggering, and execution mechanisms that are distinct from other regulated cell death pathways [1]. Currently, ferroptotic cell death can be inhibited by strategies such as using lipophilic antioxidants, employing iron chelators, directly inhibiting lipid peroxidation, or depleting polyunsaturated fatty acyl phospholipids (PUFA-PLs), which serve as key substrates driving lipid peroxidation [2,3]. Key to the lipid peroxidation cascade are lipoxygenase enzymes, particularly arachidonate lipoxygenases (ALOXs), such as ALOX15, which initiates oxidative damage by catalyzing the oxygenation of PUFAs, leading to the formation of highly reactive lipid hydroperoxides, a hallmark of ferroptosis [4]. In the central nervous system (CNS), evidence suggests that dysregulation of iron homeostasis contributes to oxidative stress that eventually drives ferroptosis [5,6], which is increasingly recognized as a major pathogenic mechanism of neurological cell death in diseases such as Parkinson’s disease (PD), Alzheimer’s disease (AD), and ischemic stroke [7,8].

Hypoxia, defined as an insufficient oxygen supply to meet cellular metabolic demands, is a prominent feature in many disease states, particularly ischemic stroke [9,10]. Cerebral microvascular endothelial cells play a crucial role in maintaining blood–brain barrier (BBB) integrity and ensuring cerebral vascular homeostasis [11]. These cells are especially vulnerable to hypoxia-induced injury and are highly susceptible to ferroptotic cell death [12], as hypoxic conditions can compromise their structural and functional integrity, disrupt vascular stability, and contribute to neural injury. Hypoxia significantly modulates ferroptotic signaling, primarily by activating hypoxia-inducible factor-1α (HIF-1α), a key transcriptional regulator of genes involved in cellular metabolism, angiogenesis, and survival [12]. This hypoxic environment often promotes the activity of key ferroptotic drivers, including ALOX15, exacerbating cellular injury. However, the impact of hypoxic stimuli on ferroptosis can be dichotomous, either suppressing or promoting it, depending on the specific cell type and microenvironmental context [12].

Dysregulated iron is involved in the generation of lipid ROS, either through Fenton chemistry or via iron-dependent oxidases [5], thereby producing highly damaging hydroxyl radicals (•OH) that initiate PUFA degradation [13,14]. Normally, cellular iron homeostasis is precisely regulated by a balance of iron importers, exporters, and storage proteins [15]. However, hypoxia-induced ferroptosis occurs through iron dysregulation, which may involve various metabolic enzymes such as prolyl 4-hydroxylase isoform 1 (PHD1) [16] and lipoxygenase (LOX) [17], for which iron serves as a cofactor in cellular oxygen sensing and lipid metabolism, respectively. The generation of toxic lipid hydroperoxides (LOOH) is often mediated by radicals, which ultimately exert the toxic effects of ferroptotic cell death [14]. GPX4 is a key antioxidant enzyme that reduces LOOH to less reactive alcohols in a glutathione (GSH)-dependent manner [18]. Notably, hypoxia impairs the GPX4–GSH axis, disrupting GPX4 activity and the GSH/oxidized glutathione (GSSG) ratio, thereby accelerating ferroptosis [19,20].

Peroxisome proliferator-activated receptor gamma (PPARγ) is a nuclear receptor central to lipid and glucose metabolism, adipogenesis, and inflammatory responses. Its activity is profoundly influenced by oxygen levels; indeed, PPARγ suppression is a consistent pathological hallmark in numerous vascular diseases exacerbated by hypoxia [21]. Endothelial PPARγ downregulation, observed in chronic intermittent hypoxia models, contributes to endothelial dysfunction and oxidative stress [22]. By regulating genes involved in lipid metabolism and fatty acid transport, PPARγ directly influences the cellular availability of PUFAs, which are prime substrates for lipid peroxidation [23,24]. Fatty acid binding protein 5 (FABP5), a critical PPARγ downstream target and cytosolic lipid chaperone, transports long-chain PUFAs. In ferroptosis, FABP5 can sensitize cells by delivering pro-ferroptotic PUFAs to peroxidation sites, increasing substrate availability for lipoxygenase enzymes [25,26,27]. Given these established influences on lipid peroxidation and its critical role in ferroptosis, there is an imperative need to investigate natural compounds as more efficient and less toxic therapeutic candidates for mitigating ferroptosis.

Luteolin (3′,4′,5,7-tetrahydroxyflavone), a natural flavonoid widely found in many vegetables and medicinal herbs, is known for its multifaceted protective properties, including potent antioxidant, anti-inflammatory, and iron-chelating activities that reduce iron available for Fenton reactions [28,29,30]. Recent studies indicate that luteolin strengthens the GPX4–GSH antioxidant system in various cell types [31] and mitigates AD pathologies by counteracting amyloid beta-induced oxidative stress and mitochondrial impairments through regulation of PPARγ activity [32]. Consequently, its anti-ferroptosis activity is attracting significant attention, given its association with ferroptosis-mediated neurodegeneration. Despite these reported antioxidant and metabolic effects, the relationship between luteolin, PPARγ/FABP5-associated lipid metabolism, and ALOX15 expression during hypoxia-induced ferroptosis in brain microvascular endothelial cells remains unclear.

Recent studies have provided direct evidence that luteolin can modulate ferroptosis in different experimental systems. Wen et al. demonstrated that luteolin attenuated radiation-induced endothelial injury by regulating the HSPB1/SLC7A11/GPX4 ferroptosis pathway in HUVECs [33]. Han et al. reported that luteolin protected against CCl_4_-induced hepatic injury by suppressing ferroptosis through regulation of SLC7A11 and associated GPX4/GSH-dependent antioxidant responses [34]. More recently, Li et al. showed that luteolin inhibited ferroptosis in cerebral ischemia–reperfusion models through the NRF2/SLC7A11/GPX4 pathway [35]. Collectively, these studies provide evidence for the anti-ferroptotic activity of luteolin but also indicate that its regulatory mechanisms vary according to cell type and pathological context.

Despite increasing evidence linking ferroptosis to hypoxia-associated neurovascular injury, the molecular mechanisms regulating ferroptotic cell death in brain microvascular endothelial cells remain incompletely understood [12,36,37]. In particular, the involvement of the PPARγ/FABP5 signaling axis in hypoxia-induced ferroptosis and lipid peroxidation has not been fully elucidated [25,26,27]. Luteolin, a naturally occurring flavonoid with potent antioxidant, anti-inflammatory, and neuroprotective properties, has been reported to modulate oxidative stress and lipid metabolism [28,30,32]; however, its protective role against hypoxia-induced ferroptosis in brain microvascular endothelial cells has yet to be clarified. Therefore, the present study investigated the effects of luteolin on cobalt chloride (CoCl_2_)-induced hypoxic injury in human brain microvascular endothelial (HBEC-5i) cells, with particular emphasis on ferroptosis-associated oxidative stress, intracellular Fe^2+^ accumulation, lipid peroxidation, GPX4–GSH antioxidant defense, and changes in the expression of PPARγ, FABP5, and ALOX15. Our findings provide further insight into the ferroptosis-associated molecular responses to luteolin in brain microvascular endothelial cells under hypoxia-mimetic stress.

Importantly, the present study extends previous reports of luteolin-mediated ferroptosis regulation, which have primarily implicated the HSPB1/SLC7A11/GPX4 and NRF2/SLC7A11/GPX4 pathways [33,34,35], by examining coordinated changes in PPARγ, FABP5, and ALOX15 expression in human brain microvascular endothelial cells under hypoxia-mimetic stress. This cell-specific and signaling context provides a distinct perspective on the potential mechanisms associated with the anti-ferroptotic effects of luteolin.

## 2. Materials & Methods

### 2.1. Cell Culture

HBEC-5i cells were obtained from ATCC (Manassas, VA, USA; CRL-3245). Cells were cultured and maintained in EndoGRO™ MV Complete Culture Media Kit from Sigma-Aldrich (St. Louis, MO, USA; SCME004) supplemented with 10% fetal bovine serum (FBS) at 37 °C in a humidified atmosphere containing 5% CO_2_. Before cell seeding, all culture vessels were pre-coated with a 0.1% sterile gelatin solution, incubated for 1 h in an incubator, and then rinsed with 1× Dulbecco’s phosphate-buffered saline (DPBS). The culture medium was replenished twice weekly to remove non-adherent cells and ensure optimal nutrient availability. Cells were subcultured using 0.25% trypsin upon reaching approximately 85% confluence. All experiments were conducted using cells at passages P4 to P10.

### 2.2. Cell Treatment

**Figure 1:**
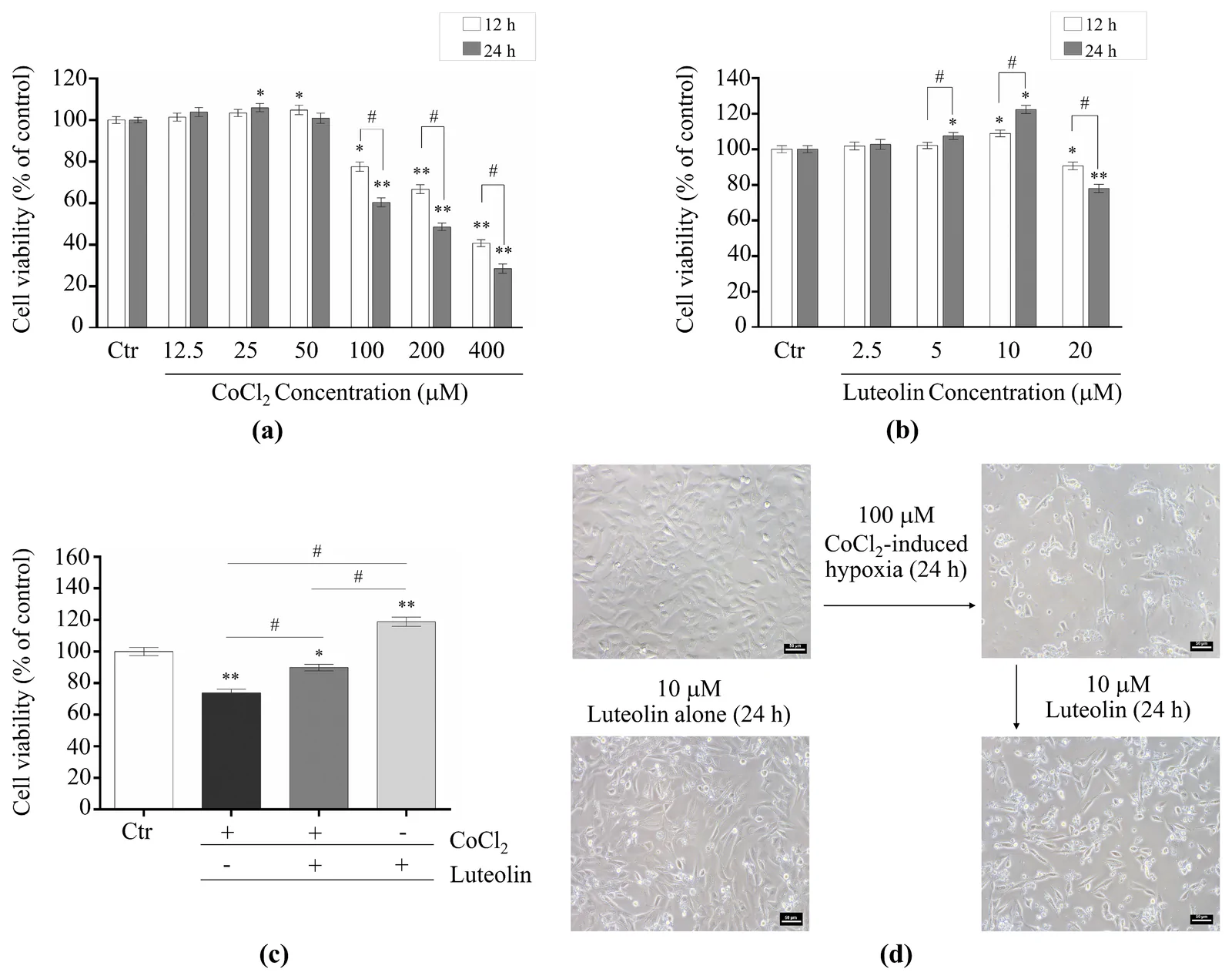
Concentration- and time-dependent effects of CoCl_2_ and luteolin on HBEC-5i cell viability. Cell viability was assessed using the MTT assay. (**a**) Cells were treated with increasing concentrations of CoCl_2_ for 12 or 24 h. (**b**) Cells were treated with increasing concentrations of luteolin for 12 or 24 h. (**c**) Experimental design showing four groups: (1) normoxia (untreated control, ctr), (2) CoCl_2_-induced hypoxia (100 μM CoCl_2_ for 24 h), (3) luteolin (10 μM) treatment under CoCl_2_-induced hypoxic conditions, and (4) luteolin alone (10 μM). (**d**) Representative images showing cell morphology under the indicated conditions. Data are presented as mean ± SEM from three independent experiments performed in triplicate. Statistical significance was determined as follows: * *p* < 0.05 and ** *p* < 0.01 vs. untreated control; ^#^ *p* < 0.05 vs. CoCl_2_-treated group.

CoCl_2_ was purchased from Glentham Life Science (Corsham, Wiltshire, UK; Catalog No.: GK0658). A 25 mM stock solution was prepared by dissolving CoCl_2_ in distilled water, filtering through a 0.22 μm membrane, aliquoting, and storing at –80 °C, as previously described [38]. To establish a hypoxia-mimetic model, cells were subjected to overnight serum deprivation followed by treatment with 100 µM CoCl_2_ for 24 h. Control cells were maintained in complete medium under normoxic conditions. Luteolin (purity ≥ 98) was obtained from Sigma-Aldrich (St. Louis, MO, USA; Catalog No.: L9283). Following hypoxia induction, cells were treated with 10 μM luteolin for 24 h. Corresponding untreated control cells were included in all experiments. The 10 μM luteolin concentration was selected based on dose–response analysis (Figure 1b) showing optimal viability enhancement at 24 h with minimal cytotoxicity, consistent with protective concentrations reported in prior endothelial and neuronal studies.

### 2.3. Cell Viability Assay

Cell viability was determined using the MTT assay (Sigma-Aldrich, St. Louis, MO, USA). Briefly, HBEC-5i cells were seeded into 96-well plates at a density of 2 × 10^4^ cells/well and allowed to adhere for 24 h. Cells were then exposed to hypoxia-mimetic conditions using various concentrations of CoCl_2_ (0, 12.5, 50, 100, 200, and 400 μM) for specified durations (12 or 24 h). Similarly, cells were treated with various concentrations of luteolin (0, 2.5, 5, 10, and 20 μM) for 12 and 24 h, respectively. Subsequently, a 2 mg/mL MTT solution was added to each well and incubated for 2 h. After incubation, the medium was gently removed, and the resulting purple formazan crystals were solubilized in dimethyl sulfoxide (DMSO). Absorbance was measured at 570 nm using a Synergy HT microplate reader (BioTek Instruments, Winooski, VT, USA). Cell viability was calculated as a percentage relative to untreated control cells.

### 2.4. Iron Content Assay

Intracellular ferrous iron (Fe^2+^) levels, a hallmark of ferroptosis-associated iron dysregulation, were quantified using a ferrous iron (Fe^2+^) colorimetric assay kit from Thermo Fisher Scientific (Waltham, MA, USA; EEA008) according to the manufacturer’s instructions. Briefly, approximately 1 × 10^6^ cells/sample were lysed in the provided lysis buffer, and debris was removed by centrifugation. The pellet was resuspended in homogenization buffer, incubated on ice, and centrifuged at 15,000× *g*. The supernatant, containing the extracted cellular Fe^2+^, was collected and incubated at 37 °C for 10 min to facilitate colorimetric reaction development. Absorbance was measured at 593 nm using a Synergy HT microplate reader (BioTek Instruments, Winooski, VT, USA), and Fe^2+^ concentrations were calculated according to the instructions.

### 2.5. Intracellular Reactive Oxygen Species (ROS) Assay

Intracellular ROS production was quantified using the 2′,7′-dichlorofluorescein diacetate (DCFDA) Reactive Oxygen Species Assay Kit (Thermo Fisher Scientific, Waltham, MA, USA). Briefly, cells were seeded in 96-well plates at a density of 2 × 10^4^ cells/well. After experimental treatments, the cell culture medium was removed, and cells were incubated with 10 µM DCFDA in phosphate-buffered saline (PBS) for 30 min at 37 °C. The fluorescence of the oxidized product, 2′,7′-dichlorofluorescein (DCF), was measured using a Synergy HT Multi-Mode Microplate Reader (BioTek Instruments, Winooski, VT, USA) at 485 nm excitation and 520 nm emission wavelengths. ROS levels were normalized to untreated controls and expressed as percentages.

### 2.6. Lipid Peroxidation Assay

Lipid peroxidation was evaluated by measuring malondialdehyde (MDA), a major end-product of oxidative lipid degradation, using a colorimetric assay based on the reaction of MDA with thiobarbituric acid (TBA). Total protein content was determined using a bicinchoninic acid (BCA) assay kit (Thermo Fisher Scientific, Waltham, MA, USA) for normalization. The formation of a stable chromophore was detected at 532 nm using a Synergy HT microplate reader (BioTek, Winooski, VT, USA). MDA levels were expressed as nmol/mg protein.

### 2.7. Glutathione Content Assay

Intracellular levels of reduced glutathione (GSH) and oxidized glutathione (GSSG) were quantified using a GSH and GSSG Assay Kit from Abcam (Cambridge, UK; ab156681) according to the manufacturer’s protocol. Standard curves for both GSH and GSSG were established using serial dilutions. Briefly, cell lysates were prepared and diluted 1:10 with the provided assay buffer, followed by incubation for 30 min at room temperature. Fluorescence intensity was measured using a Synergy HT microplate reader (BioTek, Winooski, VT, USA) at 490 nm excitation and 520 nm emission wavelengths.

### 2.8. Western Blot Analysis

Cells were lysed in RIPA buffer supplemented with 1% protease inhibitor cocktail. Lysates were then centrifuged at 2000× *g* for 15 min at 4 °C to remove cellular debris. Protein concentrations were subsequently determined using the BCA assay kit. Equal amounts of protein (40 µg per lane) were separated by 12–15% SDS-polyacrylamide gel electrophoresis and transferred onto nitrocellulose membranes (Bio-Rad, Hercules, CA, USA). Membranes were blocked for 1 h at room temperature in 5% non-fat milk prepared in Tris-buffered saline with 0.1% Tween 20 (TBS-T). After blocking, membranes were incubated overnight at 4 °C with specific primary antibodies diluted in blocking buffer. The primary antibodies included: anti-HIF-1α (1:500, #ab179483, Abcam), anti-VEGF (1:1000, #2463, Cell Signaling Technology), anti-GPX4 (1:1000, #52455, Cell Signaling Technology), anti-ALOX15 (1:1000, #ab244205, Abcam), anti-PPARγ (1:1000, #ab78920, Abcam), and anti-FABP5 (1:1000, #ab255276, Abcam). Anti-β-actin (1:5000, #ab8227, Abcam) served as the endogenous loading control. After primary antibody incubation, membranes were washed and then incubated with HRP-conjugated secondary antibodies for 2 h at room temperature. Protein bands were visualized using the SuperSignal West Pico chemiluminescence detection kit (Thermo Fisher Scientific Inc., Waltham, MA, USA) and imaged with the ChemiDoc XRS+ System. Densitometric analysis of the blots was performed using Image Lab Software (Bio-Rad, Hercules, CA, USA). Relative protein expression levels were normalized to β-actin. During preliminary optimization experiments, molecular weight markers were run alongside the samples to verify that the immunoreactive bands detected with each antibody corresponded to the expected apparent molecular weight of the respective target protein. However, images containing the molecular weight markers from these preliminary experiments were not captured and retained. The available uncropped membrane images are provided in the Supplementary Materials, and the expected apparent molecular weight of each target protein is indicated in the corresponding figure legend.

### 2.9. Statistical Analysis

All data are presented as mean ± standard error of the mean (SEM) from three independent experiments performed in triplicate. Differences among multiple groups were evaluated using one-way analysis of variance (ANOVA), followed by Bonferroni’s post hoc test for multiple comparisons. A *p*-value of <0.05 was considered statistically significant. All statistical analyses were conducted using GraphPad Prism version 8.0.2 (GraphPad Software, Inc., La Jolla, CA, USA).

## 3. Results

### 3.1. Dose- and Time-Dependent Effects of CoCl_2_ and Luteolin on HBEC-5i Cell Viability

To investigate the dose- and time-dependent effects of CoCl_2_ and luteolin on HBEC-5i cell properties, we assessed cell viability using the MTT assay. As shown in Figure 1a, CoCl_2_ exhibited both dose- and time-dependent effects on cell viability. Notably, low CoCl_2_ concentrations (12.5–50 μM) did not induce cytotoxicity over 12 or 24 h; instead, we observed a slight increase in viability. Conversely, higher concentrations (≥100 μM) led to a significant and time-dependent reduction in viability, with we observed maximal cytotoxicity at 24 h. Because 100 μM CoCl_2_ for 24 h consistently reduced cell viability by approximately 45%, we selected this concentration for subsequent mechanistic studies. In parallel, we assessed the effect of luteolin on cell viability. As shown in Figure 1a, 2.5 μM luteolin had no significant effect, whereas 5–10 μM luteolin markedly enhanced cell survival at 24 h in a time-dependent manner (Figure 1b), suggesting a direct cytoprotective or proliferative effect. However, a higher concentration of luteolin (20 μM) consistently decreased cell viability across all time points, suggesting a potential dose-dependent toxicity or a biphasic effect, a common characteristic of many bioactive compounds. Given its effect on cell viability, we selected 10 μM luteolin for 24 h as the optimal dose for further evaluation.

Furthermore, we evaluated the potential of 10 μM luteolin to mitigate 100 μM CoCl_2_-induced cytotoxicity in HBEC-5i cells. As shown in Figure 1c, luteolin significantly enhanced cell viability. Although it did not fully restore viability to the levels observed in untreated control cells, it provided a partial but significant amelioration of CoCl_2_-induced cellular damage. Subsequently, we observed the morphology and cell number under a microscope. As shown in Figure 1d, cells treated with CoCl_2_ for 24 h exhibited a substantial reduction in cell population; subsequent treatment with luteolin for 24 h promoted cell regrowth.

### 3.2. Induction of HIF-1α and VEGF Under Hypoxia-Mimetic Conditions

**Figure 2:**
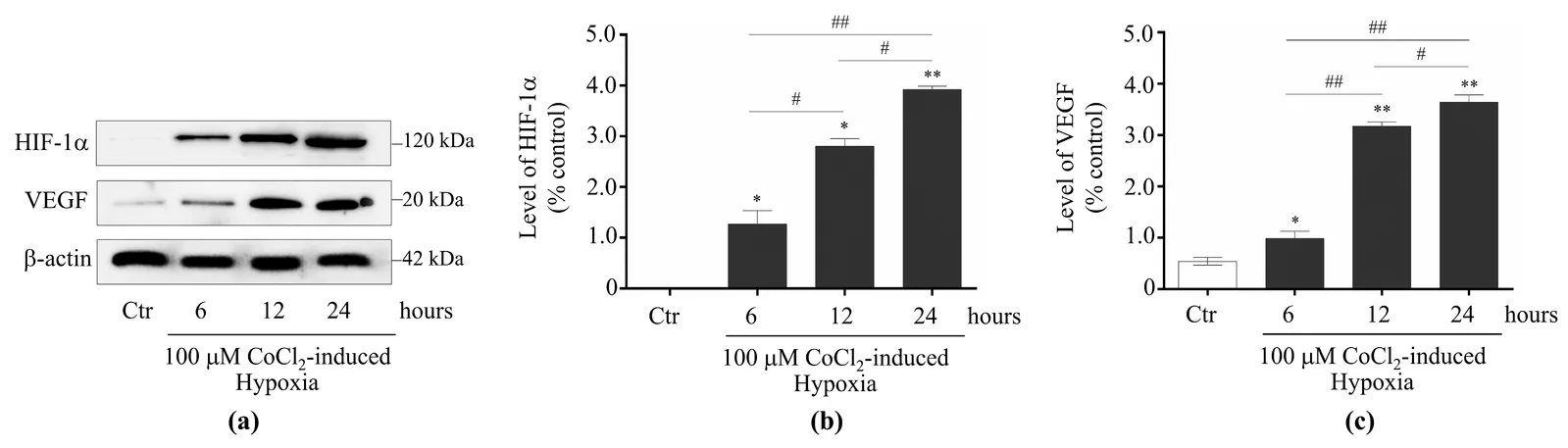
Expression of HIF-1α and VEGF under hypoxic conditions in HBEC-5i cells. Cells were exposed to 100 μM CoCl_2_ for 0, 6, 12, and 24 h. (**a**) Representative Western blot images showing the expression of HIF-1α (expected apparent molecular weight ~120 kDa) and VEGF (expected apparent molecular weight ~20 kDa), key proteins involved in cellular responses to hypoxia. The available uncropped Western blot membrane is provided in Supplementary Materials. Quantitative analysis of (**b**) HIF-1α and (**c**) VEGF protein expression was normalized to β-actin. Data are presented as mean ± SEM from three independent experiments performed in triplicate. Statistical significance was determined as follows: * *p* < 0.05 and ** *p* < 0.01 vs. untreated control; ^#^ *p* < 0.05 and ^##^ *p* < 0.01 vs. CoCl_2_-treated group.

Building on the selection of 100 μM CoCl_2_ to induce cellular stress, the molecular response of HBEC-5i cells was further investigated by assessing the protein expression of HIF-1α and its key downstream target, vascular endothelial growth factor (VEGF), over a 6- to 24 h time course. Western blot analysis revealed that HIF-1α protein expression was significantly increased in a time-dependent manner compared with untreated control cells (Figure 2a,b), confirming effective activation of hypoxia-responsive signaling pathways. Concurrently, VEGF expression also increased significantly in a time-dependent manner compared with untreated control cells (Figure 2a,c), further validating activation of the HIF-1α axis. These findings collectively demonstrate that CoCl_2_-mimicked hypoxia effectively upregulates the HIF-1α/VEGF pathway in HBEC-5i cells. This upregulation represents a crucial cellular adaptation mechanism for survival under low-oxygen conditions and promotes angiogenesis. Therefore, our CoCl_2_ model is validated as a reliable inducer of cellular hypoxic stress.

### 3.3. Luteolin Mitigates Excess Iron-Induced Oxidative Stress in Hypoxic HBEC-5i Cells

**Figure 3:**
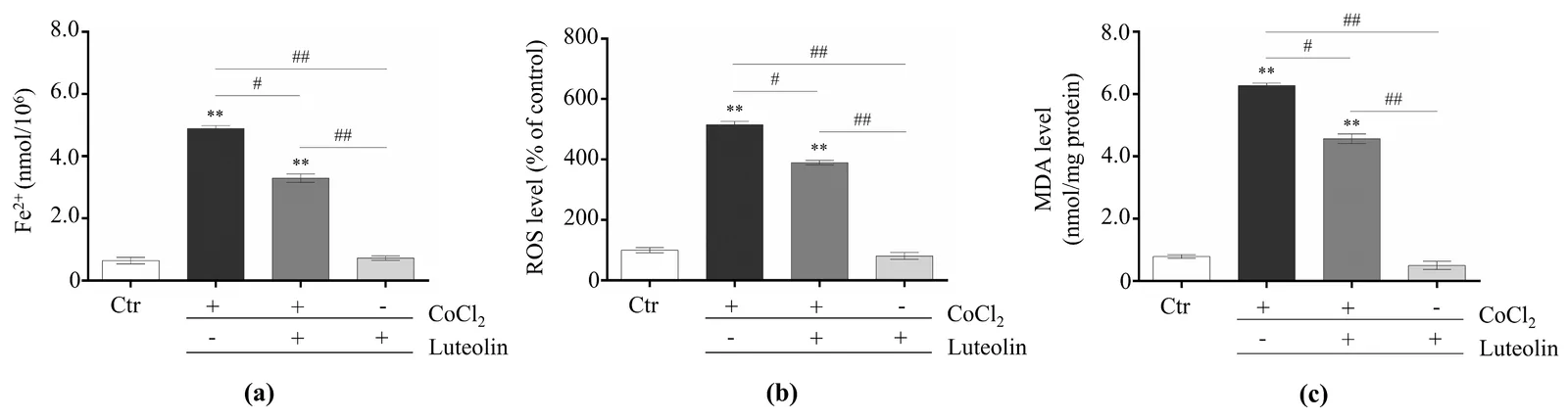
Luteolin reduces intracellular ferrous iron and oxidative stress markers under hypoxic conditions in HBEC-5i cells. Cells were exposed to 100 μM CoCl_2_ for 24 h, followed by treatment with 10 μM luteolin for 24 h. (**a**) Intracellular ferrous iron (Fe^2+^) levels were quantified using a ferrous iron colorimetric assay kit. (**b**) Intracellular reactive oxygen species (ROS) levels were measured using the DCFH-DA probe. (**c**) Malondialdehyde (MDA) levels were determined using the thiobarbituric acid (TBA) assay. Data are presented as mean ± SEM from three independent experiments performed in triplicate. Statistical significance was determined as follows: ** *p* < 0.01 vs. untreated control; ^#^ *p* < 0.05 and ^##^ *p* < 0.01 vs. CoCl_2_-treated group.

To gain insight into the molecular mechanisms underlying hypoxia-induced ferroptosis, the impact of luteolin on intracellular iron levels, a key initiating event in ferroptosis, was first assessed. Using an iron colorimetric assay kit, the results showed that CoCl_2_-induced hypoxic conditions significantly increased Fe^2+^ levels compared with untreated control cells. Importantly, subsequent treatment with 10 μM luteolin significantly decreased toxic Fe^2+^ levels. Notably, cells treated with luteolin alone exhibited intracellular iron levels comparable to those of untreated control cells (Figure 3a). CoCl_2_-induced hypoxic conditions significantly increased intracellular Fe^2+^ levels compared with untreated control cells, whereas subsequent treatment with 10 μM luteolin significantly reduced Fe^2+^ accumulation. Luteolin alone did not significantly alter intracellular Fe^2+^ levels compared with untreated control cells (Figure 3a). These findings indicate that luteolin attenuates the accumulation of intracellular ferrous iron under hypoxia-mimetic conditions. However, the present experiments do not establish whether this effect results from direct iron chelation or from changes in cellular iron uptake, storage, or export.

Given that iron overload can increase free radical production via the Fenton reaction, intracellular ROS levels and MDA content were subsequently quantified. Both are critical indicators of cellular oxidative stress and are byproducts of lipid peroxidation under hypoxic conditions. Our findings showed that CoCl_2_-induced hypoxic conditions significantly increased intracellular ROS accumulation and MDA content, consistent with oxidative stress induction. However, subsequent treatment with 10 μM luteolin significantly reduced intracellular ROS accumulation and MDA levels (Figure 3b,c), confirming its potent antioxidant and ROS-scavenging properties. Luteolin alone produced results similar to those of untreated control cells. This confirms that luteolin has protective and restorative effects against hypoxia-induced cellular damage.

### 3.4. Luteolin Enhances Antioxidant Systems in Hypoxic HBEC-5i Cells

**Figure 4:**
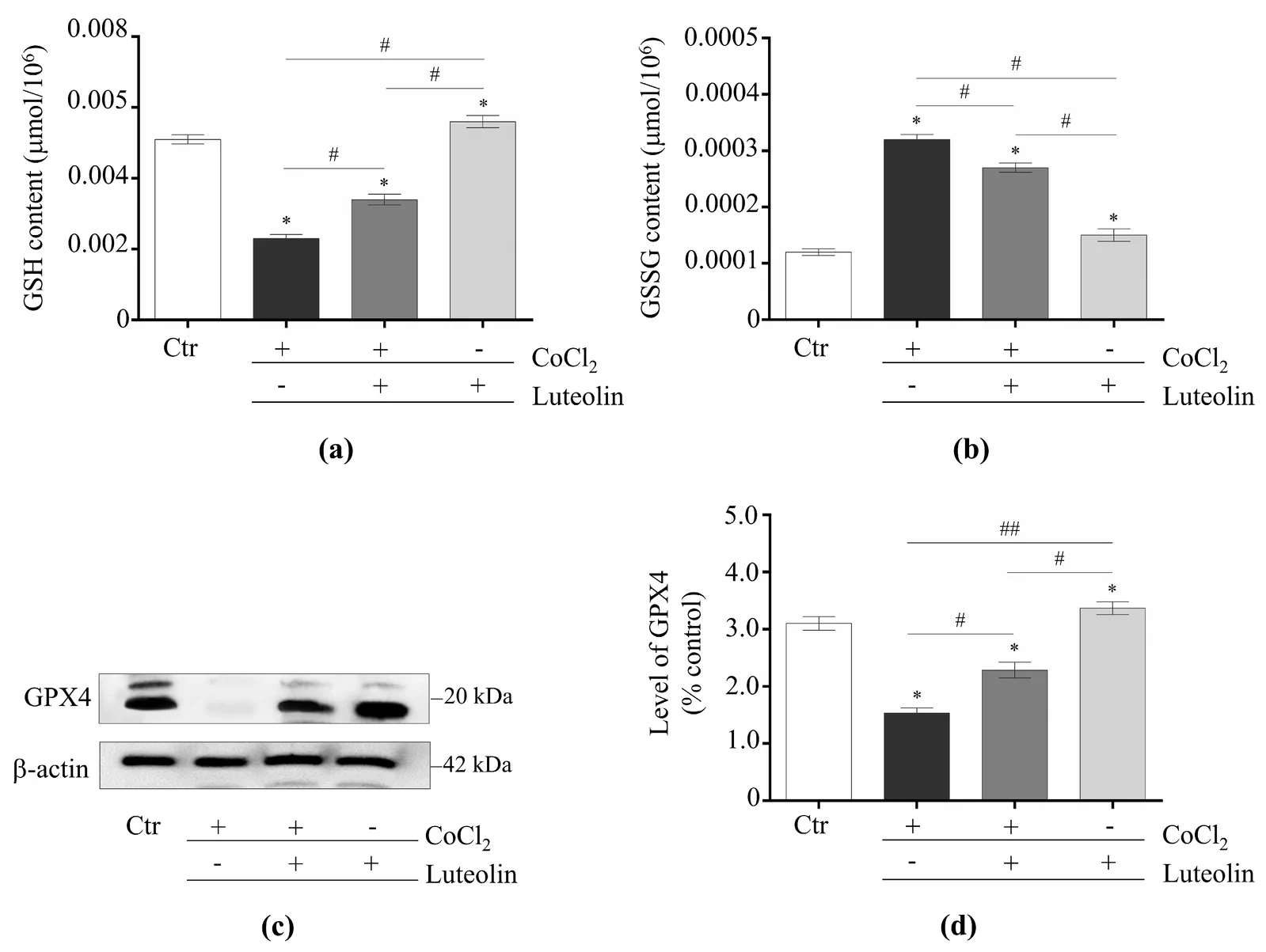
Luteolin alleviates impairment of oxidative defense systems in HBEC-5i cells under hypoxic conditions. Cells were exposed to 100 μM CoCl_2_ for 24 h, followed by treatment with 10 μM luteolin for 24 h. Quantitative analysis of (**a**) reduced glutathione (GSH) and (**b**) oxidized glutathione (GSSG) levels was performed using a GSH and GSSG assay kit. (**c**) Representative Western blot images showing GPX4 protein expression (expected apparent molecular weight ~22 kDa). The available uncropped Western blot membrane is provided in Supplementary Materials. (**d**) Quantitative analysis of GPX4 expression was normalized to β-actin. Data are presented as mean ± SEM from three independent experiments performed in triplicate. Statistical significance was determined as follows: * *p* < 0.05 vs. untreated control; ^#^ *p* < 0.05 and ^##^ *p* < 0.01 vs. CoCl_2_-treated group.

To further understand the molecular mechanisms, the critical antioxidant enzymes GSH, GSSG, and GPX4, central components of the cellular defense system against oxidative stress and key regulators of ferroptosis, were investigated. As shown in Figure 4a,b, CoCl_2_-induced hypoxic conditions resulted in a notable reduction in GSH content and a concomitant increase in GSSG content. However, subsequent treatment with 10 μM luteolin reversed these changes, leading to increased GSH content and reduced GSSG content, with results comparable to those of untreated control cells. This demonstrates luteolin’s ability to preserve cellular antioxidant capacity and mitigate ferroptotic processes by supporting glutathione homeostasis. Similarly, Western blot analysis showed that GPX4 protein expression was markedly downregulated under CoCl_2_-induced hypoxic conditions, whereas subsequent treatment with 10 μM luteolin significantly upregulated GPX4 expression (Figure 4c,d), thereby reversing these changes. These results suggest that hypoxia disrupted antioxidant defenses, while luteolin effectively restored them.

### 3.5. Luteolin Modulates ALOX15-Mediated Lipid Peroxidation and Ferroptosis-Related Proteins PPARγ and FABP5 in Hypoxic HBEC-5i Cells

**Figure 5:**
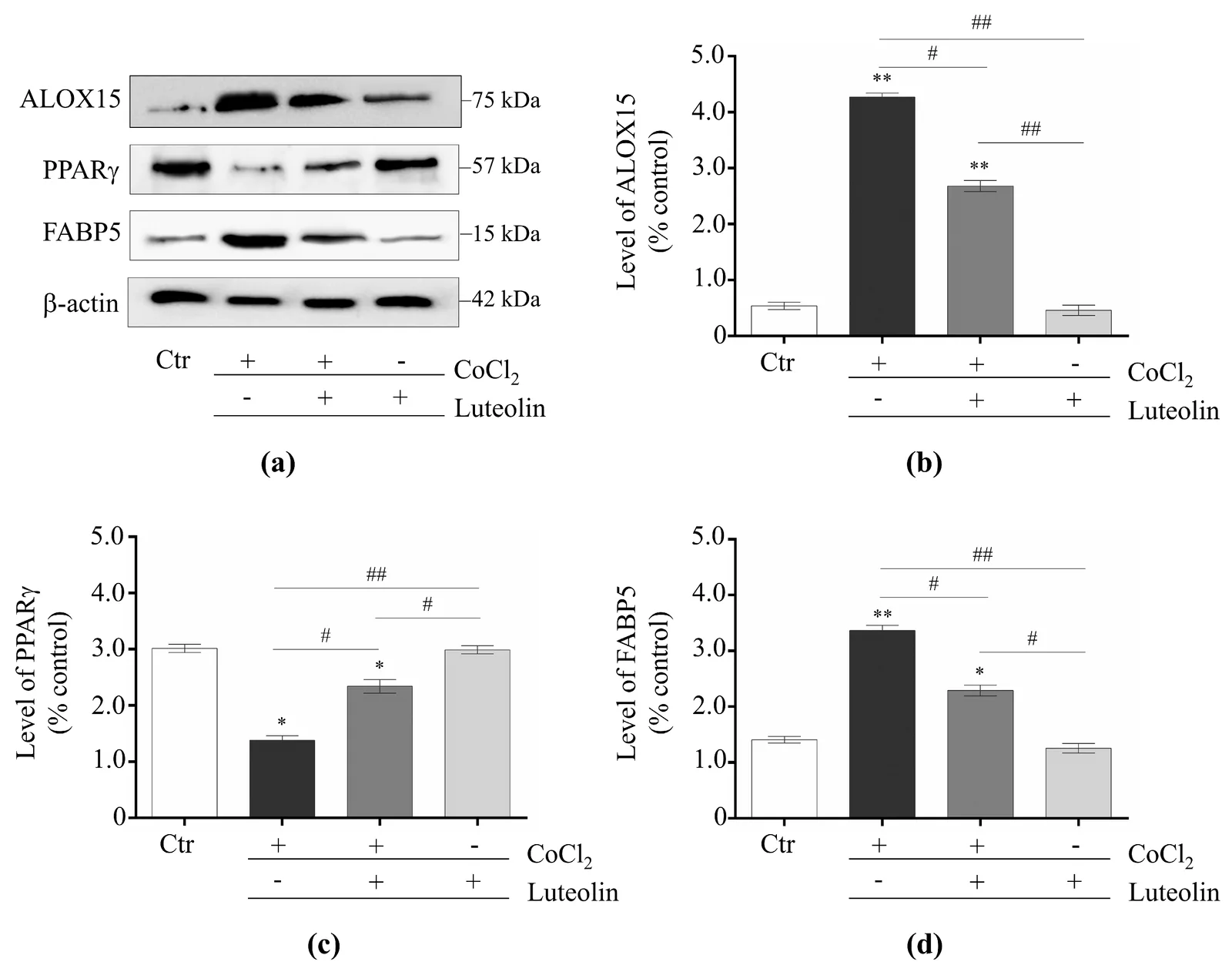
Luteolin modulates ferroptosis-related protein expression under hypoxic conditions in HBEC-5i cells. Cells were exposed to 100 μM CoCl_2_ for 24 h, followed by treatment with 10 μM luteolin for 24 h. (**a**) Representative Western blot images showing the expression of ALOX15 (a key enzyme involved in lipid peroxidation) (expected apparent molecular weight ~75 kDa), PPARγ (a nuclear receptor regulating lipid metabolism and antioxidant gene expression) (expected apparent molecular weight ~57 kDa), and FABP5 (an intracellular lipid chaperone and ferroptosis-associated biomarker). (expected apparent molecular weight ~15 kDa). The available uncropped Western blot membrane is provided in Supplementary Materials. Quantitative analyses of (**b**) ALOX15, (**c**) PPARγ, and (**d**) FABP5 expression were normalized to β-actin. Data are presented as mean ± SEM from three independent experiments performed in triplicate. Statistical significance was determined as follows: * *p* < 0.05 and ** *p* < 0.01 vs. untreated control; ^#^ *p* < 0.05 and ^##^ *p* < 0.01 vs. CoCl_2_-treated group.

Given the observed effect of luteolin on lipid peroxidation during hypoxia-induced ferroptosis, ALOX15 protein expression was investigated by Western blot analysis. The results showed that CoCl_2_-induced hypoxic conditions significantly increased ALOX15 protein expression compared to untreated control cells. Treatment with 10 μM luteolin significantly reduced ALOX15 expression (Figure 5a,b). These findings demonstrate that the reduction in lipid peroxidation following luteolin treatment was accompanied by decreased ALOX15 expression, suggesting an association between modulation of ALOX15 and the anti-ferroptotic effects of luteolin.

Next, PPARγ and FABP5, both upstream regulators of lipid metabolism involved in oxidative stress and ferroptosis, were analyzed. Western blot analysis showed that CoCl_2_-induced hypoxic conditions significantly decreased PPARγ protein expression (Figure 5a,c). This reduction in PPARγ, a nuclear receptor essential for lipid homeostasis and antioxidant gene expression, suggests a weakened cellular defense mechanism under hypoxic stress. Treatment with 10 μM luteolin significantly restored PPARγ protein levels. This restoration was accompanied by reduced oxidative stress and ferroptosis-associated changes, suggesting a potential association between PPARγ regulation and the protective effects of luteolin. The protein expression of FABP5 was assessed. FABP5, an intracellular lipid chaperone known to transport fatty acids and interact with PPARs, has recently been identified as a functional biomarker for ferroptosis under hypoxic conditions. FABP5 was significantly upregulated under CoCl_2_-induced hypoxic conditions compared to untreated control cells (Figure 5a,d). This finding is consistent with its reported increase under cellular stress and its role in mediating lipid dynamics that contribute to ferroptosis. However, this upregulation was significantly attenuated by subsequent treatment with 10 μM luteolin. The modulation of FABP5 by luteolin may indicate reduced fatty acid uptake or altered lipid signaling, both of which are crucial for controlling lipid peroxidation, a hallmark of ferroptosis.

### 3.6. Luteolin Downregulates Hypoxia-Induced HIF-1α and VEGF Expression in HBEC-5i Cells

**Figure 6:**
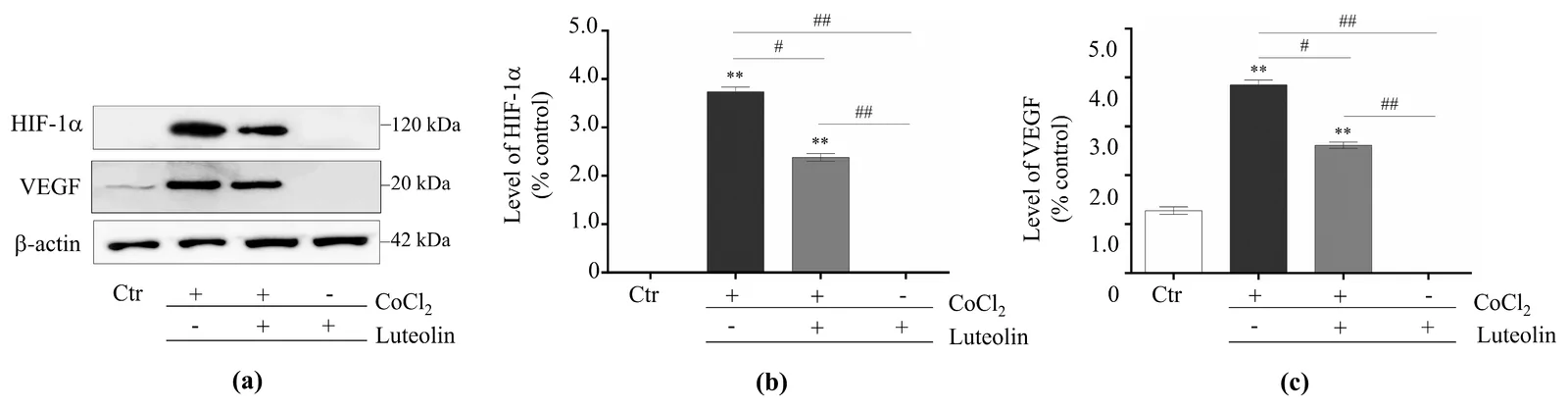
Luteolin downregulates the expression of HIF-1α and VEGF under hypoxic conditions in HBEC-5i cells. Cells were exposed to 100 μM CoCl_2_ for 24 h, followed by treatment with 10 μM luteolin for 24 h. (**a**) Representative Western blot images showing the expression of HIF-1α (expected apparent molecular weight ~120 kDa) and VEGF (expected apparent molecular weight ~20 kDa). The available uncropped Western blot membrane is provided in Supplementary Materials. Quantitative analysis of (**b**) HIF-1α and (**c**) VEGF protein expression was normalized to β-actin. Data are presented as mean ± SEM from three independent experiments performed in triplicate. Statistical significance was determined as follows: ** *p* < 0.01 vs. untreated control; ^#^ *p* < 0.05 and ^##^ *p* < 0.01 vs. CoCl_2_-treated group.

To further confirm the anti-hypoxic effects of luteolin against hypoxia-induced injury, the protein expression of HIF-1α, a master transcriptional regulator of the cellular response to hypoxia, and its downstream target responsible for angiogenesis, VEGF, was assessed by Western blot analysis. The results demonstrated that treatment with 10 μM luteolin significantly downregulated HIF-1α protein expression in hypoxic HBEC-5i cells (Figure 6a,b). Consistent with this protective role, VEGF protein expression, which was previously upregulated by hypoxia, also showed a significant reduction following luteolin treatment (Figure 6a,c). These findings indicate that luteolin effectively suppresses the core hypoxia-responsive HIF-1α/VEGF signaling pathway, thereby attenuating cellular adaptation mechanisms to low oxygen and potentially inhibiting angiogenesis. This suggests a direct molecular intervention by luteolin in counteracting the detrimental effects of hypoxia on cellular homeostasis.

## 4. Discussion

Hypoxia-induced ferroptosis has emerged as an important mechanism contributing to blood–brain barrier disruption and neurovascular injury during ischemic stroke and other neurological disorders. However, the molecular mechanisms regulating ferroptosis in brain microvascular endothelial cells under hypoxic conditions remain incompletely understood. In the present study, luteolin attenuated CoCl_2_-induced ferroptosis-associated changes in HBEC-5i cells, including disturbances in iron and redox homeostasis, lipid peroxidation, and alterations in the expression of PPARγ, FABP5, and ALOX15. While the antioxidant and anti-inflammatory properties of luteolin have been extensively reported, its role in regulating ferroptosis-associated signaling in brain microvascular endothelial cells has received limited attention. Our findings therefore provide additional insight into the molecular responses of the neurovascular endothelium to hypoxic stress and suggest an association between luteolin-mediated protection and changes in ferroptosis-related lipid metabolic pathways. The present findings should be considered in the context of previous studies demonstrating anti-ferroptotic effects of luteolin. Wen et al. reported protection against radiation-induced endothelial ferroptosis through the HSPB1/SLC7A11/GPX4 pathway [33], while Han et al. demonstrated suppression of ferroptosis in CCl_4_-induced hepatic injury through regulation of SLC7A11 and GPX4-associated antioxidant defenses [34]. Li et al. further demonstrated that luteolin attenuated ferroptosis in cerebral ischemia–reperfusion injury through the NRF2/SLC7A11/GPX4 pathway [35]. Thus, the anti-ferroptotic activity of luteolin itself is not unique to the present study. Rather, our findings extend this evidence to human brain microvascular endothelial cells exposed to hypoxia-mimetic stress and identify coordinated changes in PPARγ, FABP5, and ALOX15 expression together with restoration of the GPX4–GSH system.

Chemical hypoxia induced by CoCl_2_ is a widely established in vitro model for investigating hypoxia-associated cellular responses because it inhibits prolyl hydroxylase activity, resulting in stabilization of hypoxia-inducible factor-1α (HIF-1α). Consistent with previous reports, exposure of HBEC-5i cells to 100 µM CoCl_2_ for 24 h significantly reduced cell viability and markedly increased HIF-1α expression, confirming successful induction of hypoxic stress. Stabilized HIF-1α subsequently promotes the transcription of hypoxia-responsive genes, including vascular endothelial growth factor (VEGF), which plays a central role in angiogenesis and vascular remodeling under hypoxic conditions [39,40,41,42,43]. In agreement with these observations, we observed significant upregulation of both HIF-1α and VEGF following CoCl_2_ treatment, supporting the utility of this hypoxia-mimetic model for investigating endothelial responses to hypoxic stress. However, CoCl_2_-induced chemical hypoxia does not fully recapitulate the complex pathophysiology of physiological ischemia.

Ferroptosis is characterized by excessive iron-dependent lipid peroxidation resulting from disruption of cellular iron homeostasis and antioxidant defense mechanisms [10,44,45]. Intracellular accumulation of ferrous iron (Fe^2+^) is considered a critical initiating event because Fe^2+^ catalyzes hydroxyl radical generation through the Fenton reaction, thereby accelerating oxidative damage to membrane phospholipids [46,47]. In the present study, CoCl_2_-induced hypoxia significantly increased intracellular Fe^2+^ accumulation, consistent with previous studies demonstrating that hypoxic signaling promotes iron overload through dysregulation of iron metabolism, including enhanced expression of divalent metal transporter 1 (DMT1) and transferrin receptor 1 (TfR1), together with reduced ferroportin expression [15,48,49]. Importantly, luteolin markedly reduced intracellular Fe^2+^ accumulation, indicating partial restoration of iron homeostasis under hypoxia-mimetic conditions. Luteolin contains a catechol B-ring (3′,4′-dihydroxy configuration) capable of coordinating transition metal ions, providing a plausible structural basis for interaction with iron [50,51,52]. However, the present study did not directly demonstrate luteolin–iron complex formation. Therefore, alternative mechanisms, including altered iron uptake, storage, or export, cannot be excluded. Future studies assessing DMT1 and TfR1-mediated iron uptake, ferritin-dependent iron storage, ferroportin-mediated export, and direct luteolin–iron binding will be required to determine the mechanism responsible for the reduction in intracellular Fe^2+^. Direct comparison with established ferroptosis inhibitors and iron chelators would also help define the relative potency and mechanistic profile of luteolin.

Lipid peroxidation is a defining feature of ferroptosis and represents the primary downstream consequence of intracellular iron accumulation and oxidative stress [53,54]. Among the enzymes involved, arachidonate 15-lipoxygenase (ALOX15) has emerged as a critical mediator of phospholipid peroxidation during ferroptotic cell death. ALOX15 catalyzes the oxidation of polyunsaturated fatty acid-containing phospholipids, leading to the formation of pro-ferroptotic lipid hydroperoxides that compromise membrane integrity [19,55]. Consistent with this mechanism, CoCl_2_-induced hypoxia significantly increased MDA production and ALOX15 expression in HBEC-5i cells, indicating enhanced lipid peroxidation under hypoxic stress. Importantly, luteolin markedly reduced both MDA accumulation and ALOX15 expression, suggesting that its protective effects extend beyond direct reactive oxygen species scavenging to modulation of enzymatic lipid peroxidation pathways associated with ferroptosis. These findings suggest that reduced ALOX15 expression is associated with the attenuation of lipid peroxidation observed after luteolin treatment. However, because ALOX15 activity was not directly inhibited or genetically manipulated, a causal role cannot be established from the present data. Although MDA provided evidence of increased lipid peroxidation, measurement of 4-hydroxy-2-nonenal (4-HNE) would provide complementary information on lipid oxidative damage [56]. Future studies incorporating both MDA and 4-HNE measurements would strengthen the mechanistic characterization of ferroptosis-associated lipid peroxidation.

Overexpression of ALOX15 has been shown to enhance ferroptosis sensitivity through mechanisms distinct from direct GPX4 inhibition [57]. Maintenance of the GPX4–GSH antioxidant defense system is essential for preventing ferroptosis because GPX4 detoxifies phospholipid hydroperoxides into non-toxic lipid alcohols using glutathione as a reducing cofactor [58,59]. Consequently, depletion of intracellular GSH and impairment of GPX4 activity are widely recognized as biochemical hallmarks of ferroptotic cell death [1,60]. In the present study, hypoxic stress disrupted cellular redox homeostasis, as demonstrated by decreased GSH levels, increased GSSG accumulation, and suppression of GPX4 expression. Luteolin treatment effectively restored the intracellular GSH/GSSG balance and increased GPX4 expression, indicating recovery of antioxidant capacity under hypoxic conditions. Restoration of the GPX4–GSH system is likely to reduce phospholipid hydroperoxide accumulation and thereby interrupt the ferroptotic cascade. These findings are consistent with previous studies demonstrating that flavonoids enhance endogenous antioxidant defenses through promotion of glutathione synthesis and activation of γ-glutamylcysteine synthetase, the rate-limiting enzyme in GSH biosynthesis [61,62,63,64]. Collectively, these findings indicate that restoration of cellular redox homeostasis is associated with the protective effects of luteolin against hypoxia-induced ferroptosis-associated changes in brain microvascular endothelial cells.

The upstream regulation of lipid metabolism has recently attracted increasing attention as a determinant of ferroptosis susceptibility. PPARγ is a ligand-activated nuclear receptor involved in the regulation of lipid metabolism, mitochondrial function, inflammatory responses, and oxidative stress [65,66]. In the present study, CoCl_2_-induced hypoxia decreased PPARγ expression while increasing FABP5 and ALOX15 expression, whereas luteolin partially reversed these changes. These coordinated expression patterns suggest that the PPARγ/FABP5/ALOX15 axis may be associated with ferroptosis regulation in hypoxic brain microvascular endothelial cells. Because FABP5 facilitates intracellular transport of long-chain fatty acids and polyunsaturated fatty acids that can serve as substrates for lipid peroxidation [25,67], reduced FABP5 expression may contribute to lower substrate availability for ALOX15-associated lipid oxidation. However, the present study did not establish a direct causal hierarchy among PPARγ, FABP5, and ALOX15. Future studies using PPARγ agonists or antagonists, gene-silencing approaches, and direct functional assessment of FABP5 and ALOX15 will be required to determine whether this pathway is mechanistically involved in luteolin-mediated protection. Because ACSL4 is a key determinant of ferroptosis susceptibility through enrichment of oxidizable PUFA-containing phospholipids [68], future studies should also evaluate whether luteolin-induced changes in PPARγ are accompanied by altered ACSL4 expression or activity. In addition, direct comparison of luteolin with established PPARγ agonists, including glitazones, would help determine whether the observed effects on ferroptosis-associated lipid metabolism are related to PPARγ activation and whether ACSL4 is involved in this response.

However, cell viability and molecular endpoints alone do not define endothelial barrier function. Future studies will evaluate barrier-relevant functional readouts (e.g., transendothelial electrical resistance (TEER), paracellular permeability to dextrans, and localization/expression of tight junction proteins such as ZO-1 and occludin) in multicellular BBB models to confirm whether luteolin preserves endothelial barrier integrity under hypoxia. Because DCFDA is a nonspecific ROS indicator [69], the present study does not identify the precise source of ROS. Mitochondrial electron transport, NADPH oxidases, iron-dependent reactions, and lipoxygenase-mediated processes may all contribute and should be distinguished in future studies.

From a translational perspective, luteolin possesses several characteristics that support its potential as a neurovascular protective agent. As a lipophilic flavonoid, luteolin is capable of crossing the blood–brain barrier [70], enabling direct interaction with brain microvascular endothelial cells and other components of the neurovascular unit. In the present study, luteolin significantly suppressed hypoxia-induced HIF-1α and VEGF expression, indicating attenuation of hypoxia-responsive signaling pathways associated with endothelial dysfunction and vascular remodeling. Previous studies have demonstrated that luteolin inhibits MAPK- and STAT3-dependent signaling pathways involved in HIF-1α stabilization and VEGF expression [71,72]. Together with its effects on iron homeostasis, lipid peroxidation, and antioxidant defense, these findings suggest that luteolin exerts coordinated protective actions against multiple pathological processes contributing to hypoxia-induced endothelial injury. Because disruption of endothelial integrity is an early event in ischemic stroke and several neurodegenerative disorders, modulation of ferroptosis-associated signaling pathways may represent a potential target for further preclinical investigation aimed at preserving neurovascular function under pathological hypoxic conditions.

Luteolin may act as a multifaceted ferroptosis-modulating compound through both direct chemical and indirect cellular mechanisms. Its polyphenolic structure is consistent with antioxidant and potential metal-chelating properties [73], whereas the present data also show restoration of PPARγ and GPX4 together with suppression of FABP5 and ALOX15 expression. However, the current experiments do not distinguish direct radical-scavenging or iron-binding effects from indirect pathway-mediated mechanisms. Future studies incorporating direct iron-binding assays, radical-scavenging measurements, pathway-specific pharmacological modulation, and gene-silencing approaches will be required to determine the relative contribution of these mechanisms.

Several limitations should be acknowledged. First, the study was conducted using an in vitro HBEC-5i monoculture system, which does not reproduce the multicellular complexity of the neurovascular unit. Second, CoCl_2_-induced chemical hypoxia does not fully reproduce physiological oxygen deprivation or ischemia/reperfusion. Third, although multiple ferroptosis-associated endpoints were assessed, including Fe^2+^ accumulation, ROS, MDA, GSH/GSSG, GPX4, and ALOX15, pathway-specific ferroptosis inhibitors and genetic interventions were not included. Fourth, the present data demonstrate coordinated changes in PPARγ, FABP5, and ALOX15 expression but do not establish a causal signaling hierarchy. Comparison with established PPARγ agonists, including glitazones, and assessment of ACSL4 expression or activity would further clarify the involvement of PPARγ-associated lipid metabolism in the observed effects of luteolin. Fifth, the mechanisms responsible for the reduction in intracellular Fe^2+^ remain to be determined, including possible effects on iron chelation, uptake, storage, and export. Sixth, additional lipid-peroxidation markers such as 4-HNE were not measured, and their inclusion in future studies would further strengthen the characterization of ferroptosis-associated lipid peroxidation. Finally, cell viability and molecular endpoints do not directly establish preservation of endothelial barrier function; therefore, future studies should incorporate TEER, permeability assays, tight-junction proteins, physiologically controlled hypoxia, multicellular BBB models, and in vivo ischemia models.

## 5. Conclusions

Luteolin attenuated CoCl_2_-induced ferroptosis-associated changes in HBEC-5i cells, as evidenced by reduced intracellular Fe^2+^ accumulation, oxidative stress, and lipid peroxidation together with restoration of the GPX4–GSH antioxidant defense system. These protective effects were accompanied by restoration of PPARγ expression and suppression of FABP5 and ALOX15, suggesting that modulation of lipid-metabolic signaling may contribute to the anti-ferroptotic effects of luteolin. In contrast to previous studies focusing primarily on HSPB1/SLC7A11/GPX4- or NRF2/SLC7A11/GPX4-related mechanisms, the present findings extend the understanding of luteolin-mediated ferroptosis regulation to human brain microvascular endothelial cells under hypoxia-mimetic stress. Further pathway-specific and in vivo studies are required to establish causality and translational relevance.

## Data Availability

The data used to support the findings of this study are available from the corresponding authors upon request.

## Supplementary Materials

The following supporting information can be downloaded at: https://www.mdpi.com/article/10.3390/biomedicines14091981/s1, Original, uncropped Western blot images for the proteins analyzed in this study.
